# Enhancing Year-Round Cassava Production and Processing in Colombia Through Varieties with Stable Root Dry Matter Content

**DOI:** 10.3390/plants15030489

**Published:** 2026-02-05

**Authors:** Amparo Rosero, Jorge-Iván Lenis, Rommel León, Hernando Araujo, Jorge García, Alfonso Orozco, Remberto Martínez, Martha Montes, Víctor De la Ossa, Carina Cordero, Sandra Salazar, Nelson Morante, Luis-Fernando Delgado, Hernán Ceballos

**Affiliations:** 1 Centro de Investigación Obonuco, Corporación Colombiana para la Investigación Agropecuaria-AGROSAVIA, Km 5 via Pasto-Obonuco, Pasto 520038, Colombia; 2 CGIAR Research Program on Roots Tubers and Bananas (RTB), The Alliance of Bioversity International and the International Center for Tropical Agriculture (CIAT), Cali 763537, Colombialfdelgadom@usbcali.edu.co (L.-F.D.);; 3 Centro de Investigación Caribia, Corporación Colombiana para la Investigación Agropecuaria-AGROSAVIA, Zona Bananera 478037, Colombia; 4 Centro de Investigación Turipana, Corporación Colombiana para la Investigación Agropecuaria-AGROSAVIA, Kilómetro 13, Vía Montería-Cereté, Montería 230550, Colombia; 5 Centro de Investigación Motilonia, Corporación Colombiana para la Investigación Agropecuaria-AGROSAVIA, Kilómetro 5 vía a Becerril, Agustín Codazzi 202050, Colombia

**Keywords:** DMC stability, production seasonality, processing efficiency, economic impact

## Abstract

Cassava is an economically important crop in Colombia, particularly along the Caribbean Coast, where major processing industries are located. Seasonality in cassava production poses a major challenge for both industry and farmers, as current commercial varieties exhibit a pronounced decline in dry matter content (DMC) when harvest is extended beyond 10–12 months after planting (MAP). To address this issue, several experimental genotypes and three commercial checks were evaluated in multi-location trials across the Caribbean Coast under several harvest ages and specially after 10, 14, and 18 MAP. Genotype SM2828-28 emerged as a promising candidate due to its adequate sprouting, plant height, first branching height, fresh root yield, and low susceptibility to root rot and lodging. A key advantage of this clone is the stability of its DMC across different harvest ages. Extending the harvest period with appropriate germplasm may increase farmers’ income and reduce the downtime of processing facilities caused by seasonal production gaps. The evidence also suggests that DMC stability is under genetic control, indicating that it can be effectively improved through targeted breeding. However, research involving extended harvest intervals poses considerable logistical challenges.

## 1. Introduction

Cassava (*Manihot esculenta* Crantz) is a cornerstone of the global bioeconomy and ranks as the fourth most important staple crop after maize, wheat, and rice. It provides food security for more than 800 million people worldwide [1,2,3,4]. In 2022, global cassava root production reached approximately 330 million tons [5]. In 2023, Colombia harvested more than two million tons of cassava from about 210,000 hectares. The Caribbean region contributes a major share, with over 100,000 hectares under cultivation—representing 55% of national production [6]. This region hosts three large processing facilities dedicated to native starch extraction and more than 60 rural enterprises, known as “rallanderías,” which produce sour cassava starch [7].

Genetic improvement has substantially increased productivity, root and starch quality, nutritional value, and resistance to biotic and abiotic stresses [8]. In Colombia, cassava research and breeding have strategically targeted specific regions to maximize the crop’s impact. In 2017, the Colombian Corporation for Agricultural Research (Agrosavia) released three varieties adapted to this region [9]. Significant progress has also been made in enhancing nutritional quality through increased carotene content [10,11].

The identification of superior genotypes is affected by genotype-by-environment (G × E) interactions [12,13], highlighting the need to evaluate and select cassava genotypes across diverse environments to ensure the stability of key traits [9,14,15]. DMC in cassava roots is largely governed by additive genetic effects, making the development of cultivars with adequate DMC under standard harvest conditions relatively straightforward [8,16,17]. However, combining high FRY and DMC in the same genotype is more challenging due to genetic and physiological constraints [8,18,19,20]. Cultivars that achieve high FRY but exhibit low or unstable DMC are frequently rejected by industry and ultimately discarded [8].

Cassava is normally harvested near the end of the dry season, when DMC reaches its maximum [7]. However, this condition results in production peaks and seasonality in raw material production. Delaying harvest into the onset of the rainy season triggers starch remobilization from the roots to support shoot regeneration, causing a substantial reduction in root starch content [21]. Thus, starch remobilization affects directly the quality of cassava roots, a raw material for starch extraction, by reducing DMC and reducing income as industry payments are linked to product quality [22]. This phenomenon imposes two major limitations on cassava production regarding:

*Climate change*: Unexpected events such as untimely rains—now increasingly common due to climate change—disrupt normal production schedules. As a result, the quality of the roots at harvest, particularly DMC, becomes unpredictable [7].

*Seasonality of crop production*: Starch production in the Caribbean region of Colombia is strongly affected by the seasonal concentration of cassava harvesting, which occurs mainly within a four-month window (December to March). Consequently, starch processing facilities remain idle for long periods.

Cassava breeding programs at AGROSAVIA and CIAT maintain close engagement with actors across the processing and marketing value chains to ensure that breeding objectives align with industry needs [23]. Efforts to extend the harvest period and mitigate production seasonality in Colombia have revealed an unexpected limitation of current commercial varieties (described in more detail in this study), although DMC declines—as expected—after the onset of the rainy season, it does not subsequently recover. In this context, the challenge for improved cassava varieties is not combining high FRY and DMC but also maintaining DMC consistently over time. Stable DMC would offer producers greater confidence in securing favorable prices for their roots and enable processors to achieve higher production efficiency [9].

This study aimed to identify cassava genotypes suitable for extended harvest periods, thereby reducing production seasonality on the Colombian Caribbean Coast. The study was based on the hypothesis that high and stable DMC are under genetic control and therefore amenable to improvement through breeding. Ultimately, this approach could help expand production seasons, benefiting both processors and farmers.

## 2. Materials and Methods

### 2.1. Plant Material

The initial evaluation included 15 experimental clones harvested between 5 and 18 MAP in two cycles of selection, along with four commercial varieties, Corpoica-Verónica, Corpoica-Tai, Corpoica-Ginés, and ICA-Costeña (Table 1). Most experimental materials originated from open pollinations (SM families), for which only the female parent is known. Two clones were derived from controlled crosses (GM families), allowing both parents to be identified (Table 1).

### 2.2. Locations

Cassava genotypes were evaluated in locations across the dry and sub-humid regions of the Colombian Caribbean coast during crop cycles from 2010 to 2015 (Table 2). This initial evaluation examined different harvest times to assess the behavior and responses of multiple genotypes under varying environmental conditions across these regions, including early (5 MAP) until extended harvest time (18 MAP). As part of the breeding pipeline, uniform yield trials (UYT) were conducted in 2014 and 2015 using the fourteen selected genotypes in Ciénaga de Oro and Cereté (Córdoba); Corozal, La Unión, and San Antonio de Palmito (Sucre); and Santo Tomás (Atlántico). Based on their agronomic performance, six genotypes were selected and subsequently evaluated during 2017–2019. This stage, referred to as the Agronomic Evaluation Test (AET), was carried out in Sabanagrande, Sabanalarga, and La Unión (Table 2). Harvest times were selected according to differences observed under previous evaluations, where 10 MAP fixed a normal harvest time, 14 MAP a period immediately rain arrived, and 18 MAP to measure a recovery time after rain.

### 2.3. Experimental Design

Genotypes were evaluated in two stages of a conventional breeding scheme: Uniform Yield Trials (UYT, also referred to as multi-location trials) and Agronomic Evaluation Trials (AET), both conducted in accordance with Colombian regulations for the development of new varieties [9,14]. UYTs were established using a randomized complete block design (RCBD) with three replications. Each plot consisted of five rows, each five meters long, with a planting density of 12,500 plants per hectare (1 m between rows and 0.9 m within rows), totaling 25 plants per plot. AETs followed the same RCBD structure but with four replications, using the same row number, row length, and planting density. Fertilization and agronomic management were based on soil analysis for each location and aligned with conventional farmer practices.

Given that the breeding objective was to enable extended harvests, trials were designed for harvesting at 10, 14, and 18 MAP. Sprouting and initial vigor were assessed during the first four months. At each harvest time, plant architecture and planting material traits were evaluated one day before root extraction. Plant type was scored using a visual scale (PTS): (1) clearly better than average; (2) slightly better than average; (3) average; (4) slightly worse than average; and (5) clearly worse than average (as described previously by [9]. Total and commercial root weights per plot were recorded to estimate total and commercial yields. Normally, commercial roots have a diameter between 4 and 6 cm, and length more than 20 cm, these commercial roots are weighted, and the weight of all produced roots are registered as total root weight. Root rot incidence was assessed using a 1–5 scale, where 1 indicates no symptoms, 2 indicates moderate symptoms, 3 indicates severe symptoms, 4 indicates very severe symptoms, and 5 indicates extremely severe symptoms. DMC was determined using the gravimetric method [24].

### 2.4. Phenotypic Selection Index

Varietal ideotypes are selected based on their superiority for key traits relative to the population average. A selection index (SI) is routinely used to simultaneously account for multiple traits in the breeding process [14]. In this study, the SI incorporated FRY, DMC, and PTS, with harvesting times and locations treated as multi-environment conditions. The SI was defined as a linear combination of two or more phenotypes, weighted according to their relevance, economic value, or breeding objectives, and was computed using the CropInd script, a suitable tool for genotype selection under multi-trait and multi-environment data structures [9].

### 2.5. Statistical Analysis

The analyses of variance were conducted using a multi-location block design with a split-plot arrangement. Location (*L*_i_) represented the contrasting environmental effect, the main plot was defined by the cassava genotype (*G_k_*), and the subplot by the effect of months after planting (MAP) (*D_l_*), with block (*B_j_*) included as a random effect. Variability was partitioned to determine the significance of each factor through a combined analysis of variance, following the model described below:

$$\begin{array}{c} y_{ijklm}=L_{i}+B_{j}(L_{i})+G_{k}+{\left(L*G\right)}_{ik}+{\left(L*B*G\right)}_{ijk}+D_{l}\\ \;\;\;\;\;+{\left(L*D\right)}_{il}+{\left(G*D\right)}_{kl}+{\left(L*G*D\right)}_{ikl}+e_{ijklm} \end{array}$$

The interaction among location, block, and genotype defined the error term for the main plot and location effects, while $e_{ijklm}$ represents the experimental error of the design. Statistical analyses were performed in R [25], using the *LSD.test* function from the Agricolae package, with a significance level of *p* < 0.05 and Bonferroni adjustment for multiple comparisons. Graphs generated with ggplot2 were used to visualize differences between clones, evaluation locations, harvesting times, and their interactions, consistently with previous studies [26,27,28]. Standard errors are shown in the figures as error bars.

## 3. Results

### 3.1. Initial Evaluation of 15 Experimental Genotypes

**Figure 1:**
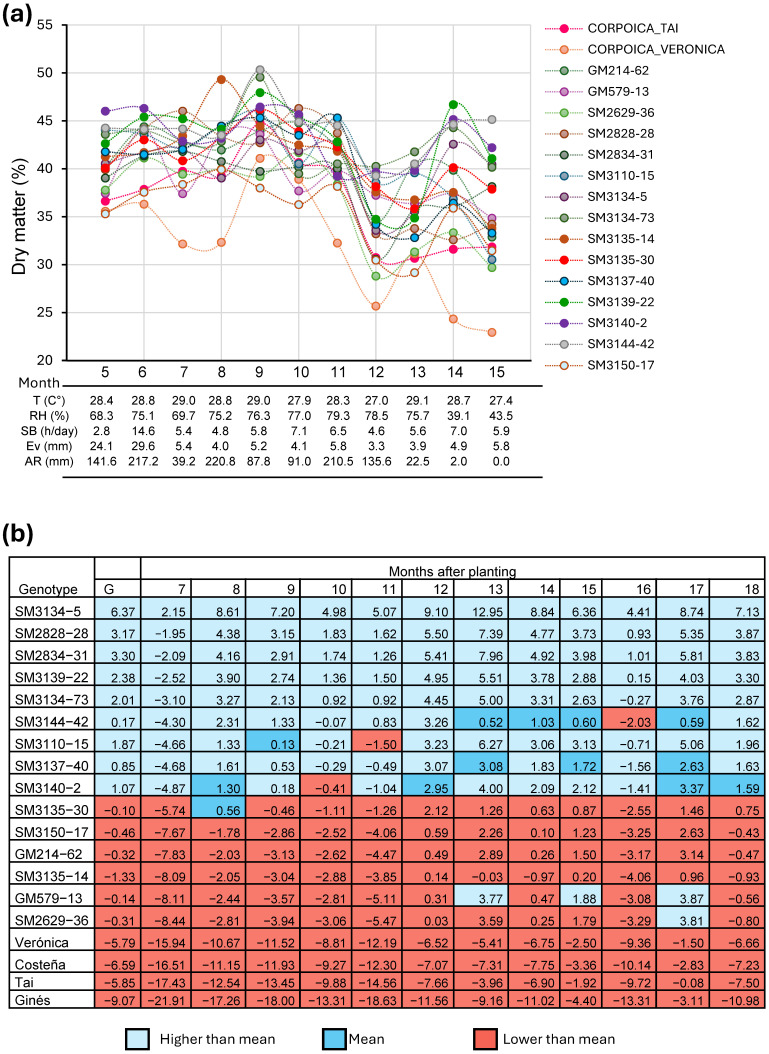
Response of dry matter in cassava roots among different harvesting times. (**a**) Dry matter stability in cassava genotypes evaluated from 5 to 15 months after planting. (**b**) Selection index calculated in genotypes evaluated at different ages after planting. T: Temperature, RH: Relative humid, SB: Solar bright, Ev: Evaporation, AR: Accumulated rainfall.

In this study, genotypes evaluated under delayed harvest conditions showed a sharp decline in DMC when plants were harvested after the onset of the first rains following the dry season. However, this reduction was not consistent across genotypes. Several clones exhibited a partial or full recovery of DMC within 1–2 months after the rains began, although the speed and extent of recovery varied (Figure 1a). Genotypes such as SM-5, SM-31, SM-28, SM-73, SM-22, and SM-15 rapidly returned to commercial DMC levels, demonstrating a clear advantage for delayed harvests at ≥15 MAP, an ability not observed in the commercial check varieties.

A selection index incorporating FRY, PTS, and DMC identified genotypes SM-5, SM-31, SM-28, SM-73, SM-22, and SM-15 as the top performers across multiple harvest ages (Figure 1b). These genotypes consistently showed excellent FRY and PTS, and—critically—exhibited both high DMC and good DMC recovery under extended harvest conditions. Consequently, these experimental clones were advanced to multilocation testing as part of the Agronomic Evaluation Test (AET).

The best-performing commercial check, Tai, ranked near the bottom of the selection index along with the other checks, Verónica, Ginés, and Costeña. Although Tai showed good FRY and excellent PTS, its DMC was generally low and failed to recover under extended harvest conditions (Table 3). Reflecting the commonly observed negative relationship between DMC and FRY, clone SM-42 achieved the highest average DMC (35.25%) but produced a relatively low FRY (14.82 t/ha). In contrast, genotypes SM-5 and SM-31 were among the top five performers for both traits.

### 3.2. Relationship Between Morpho-Agronomic Variables in Six Cassava Genotypes

**Figure 2:**
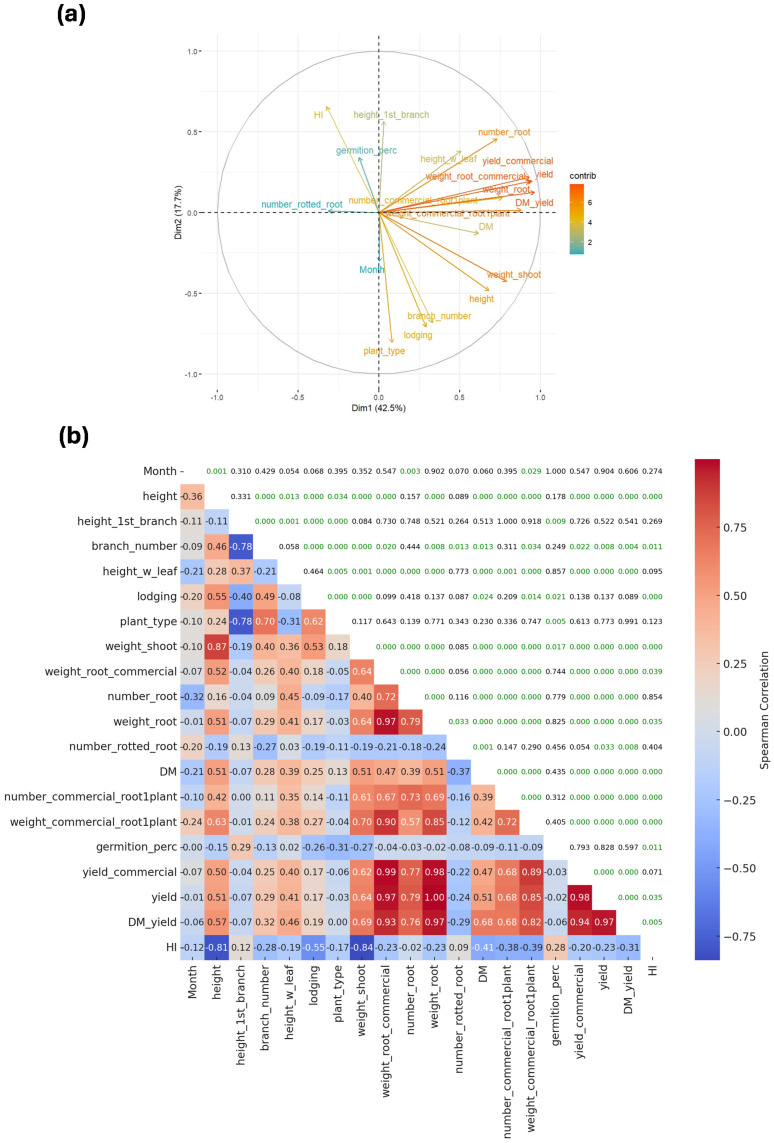
Relationship among morphoagronomic traits. (**a**) Principal component analysis and (**b**) Spearman correlation in agronomic variables in cassava. Spearman’s correlation values were calculated using *n* = 216 observations. Green color in 2b denotes significant association determined by the *p* < 0.05.

A multivariate analysis revealed that the first principal component accounts for 42.5% of the total variation, while the second explains 17.7%. Together, these two principal components capture more than 50% of the overall variability, allowing for effective dimensionality reduction while retaining most of the relevant information (Figure 2a).

Plant height, height of the first branch, and the number of branching events are key components of the PTS and contribute substantially to the variability captured by the first principal component (Figure 2a). PTS showed strong correlations with both the height of the first branch (r = –0.78) and the number of branching events (r = 0.70). Because lower PTS values indicate better plant architecture, the direction of these correlations is consistent with expectations. As anticipated, the height of the first branch was negatively correlated with the number of branching events and plant type (r = –0.78 for each; Figure 2b). Above-ground biomass (denoted as Weight Shoot in Figure 2b) was positively associated with plant height (r = 0.87). A high first branching height combined with a reduced number of branching events (ideally none) is desirable, as it promotes an erect plant architecture. However, in some cases this erect phenotype can result in excessively tall plants, increasing susceptibility to lodging (r = 0.55). Plant height also showed a negative correlation with harvest index (r = –0.81), indicating that beyond a certain point, taller plants become less efficient in converting biomass into usable yield.

Root yield was consistently associated with its main components, including total and commercial root weight and the number of roots per plant. It was also positively correlated with DMC and DRY. Interestingly, leaf retention (height_w_leaf in Figure 2b) was linked to both yield and DMC-related traits, suggesting that plants with greater foliar biomass tend to exhibit higher FRY, DRY, and DMC. Optimal partitioning requires intermediate harvest index (HI) values to maximize both FRY and DRY. The present study corroborates the limited usefulness of HI beyond the single-row stage of selection (Figure 2b).

Elevated levels of root rot pose a major concern for the feasibility of extended harvests. In this study, the number of rotted roots was generally negatively correlated with FRY and DMC (Figure 2b). However, these correlations were weak to moderate, indicating that, at least under the environmental conditions evaluated, root rot may not be a critical constraint to achieving maximum yield potential.

The month variable (e.g., age of plants at harvest) represents the time at which measurements were taken. Its positive correlations with plant height, lodging, root rot, and commercial root weight per plant were expected. These relationships indicate that plants continued growing over time, which in turn increased the likelihood of lodging. Likewise, root yield (e.g., commercial root weight) increased as harvest was delayed; however, the number of rotten roots also rose, leading to a reduction in the total number of roots (r = −0.32). DMC also showed a negative correlation with delayed harvest; however, this decline did not appear to significantly affect yield or harvest index. Finally, the strong correlations observed among yield and quality traits, including yield components, DMC, and DRY, highlight their importance as key variables for evaluating production from an economic standpoint.

### 3.3. Morpho-Agronomic Response of Six Experimental Genotypes Under Delayed Harvest

The analysis of variance revealed significant effects for all sources of variation (Table 4). Plant height, height of first branching, and stakes per plant were primarily influenced by location and genotype, with age at harvest contributing to a lesser extent.

**Figure 3:**
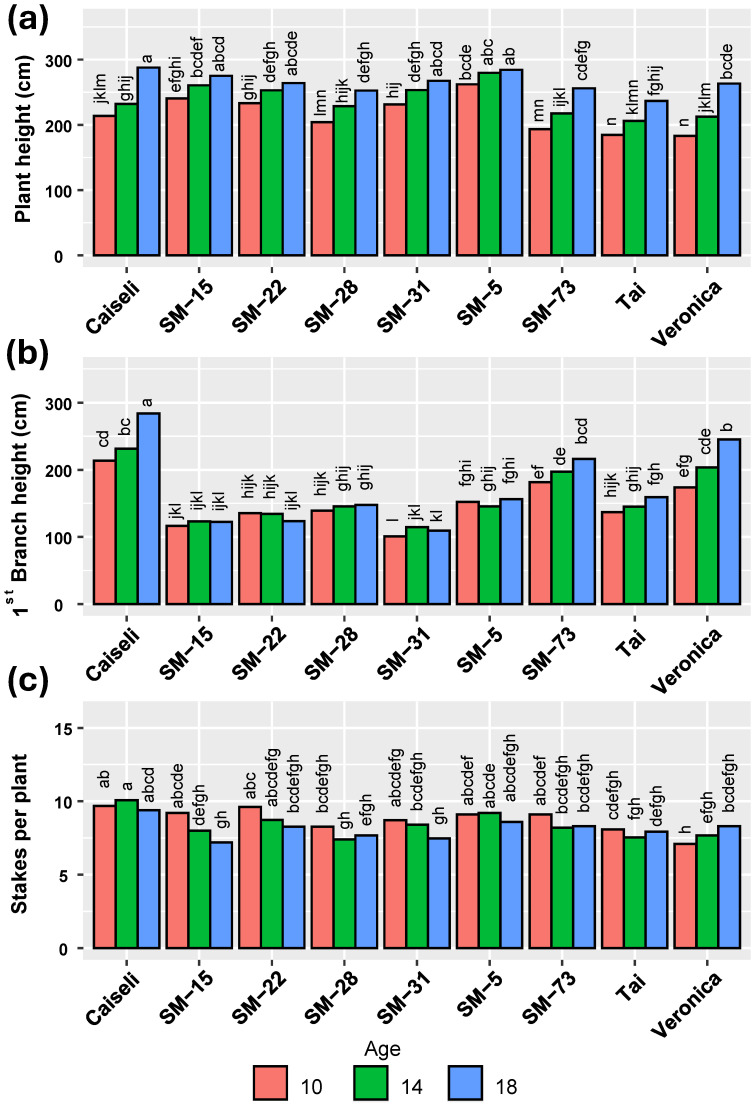
Influence of harvesting times on plant architecture of the genotypes evaluated. (**a**) Plant height, (**b**) First branch height, (**c**) Number of cuttings per plant. Means followed by a common letter (within each location) are not significantly different according to the Duncan test (α < 0.05).

During the extended harvest, plants continued to grow, reaching their maximum plant height and first branching height at 18 MAP. By this time, however, the quality of planting material had declined, which explains why a greater number of stakes per plant was recorded at 10 MAP (Figure 3). Overall, all genotypes—including the commercial checks—reached an average plant height of around 200 cm at 10 MAP (Figure 3a). Even at this early stage, all genotypes exhibited a first branching height above 100 cm (Figure 3b). In contrast, differences among genotypes in the number of stakes produced per plant were less pronounced than those observed for plant height and first branching height.

**Figure 4:**
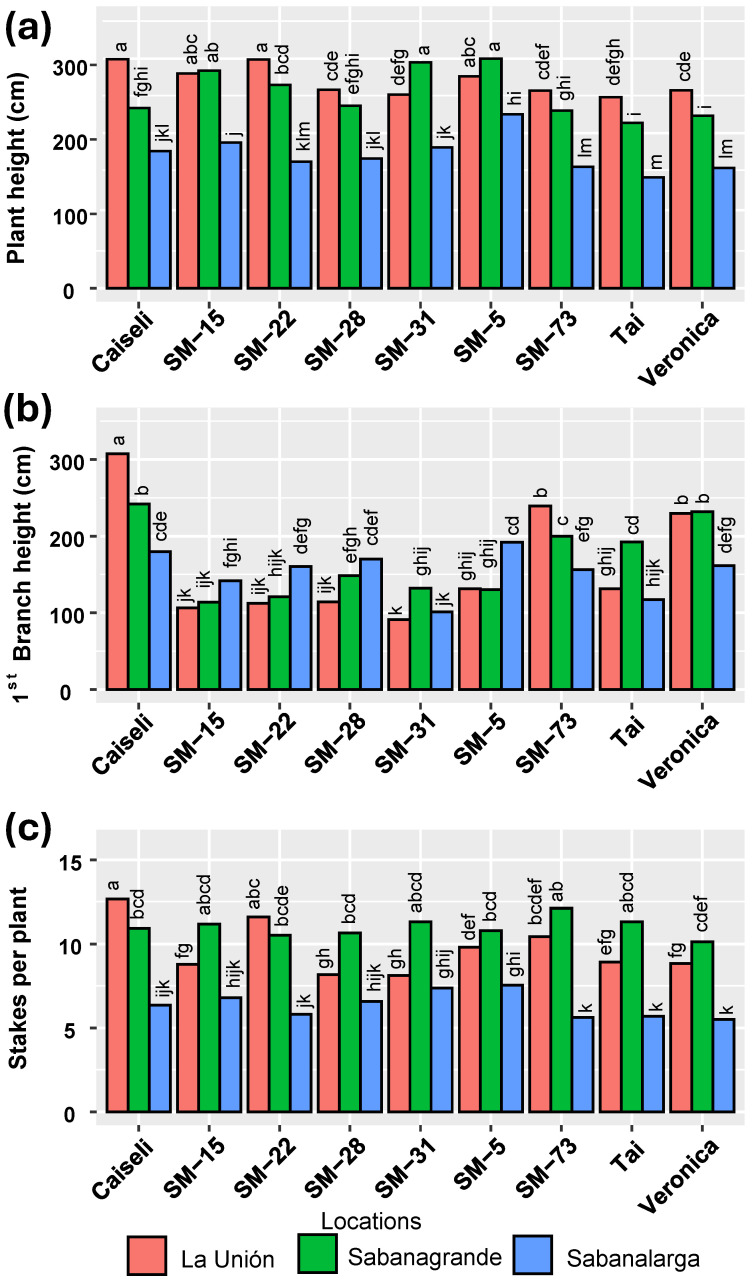
Influence of environment (locations) on plant architecture of the genotypes evaluated. (**a**) Plant height, (**b**) First branch height, (**c**) Number of cuttings per plant. Means followed by a common letter (within each location) are not significantly different according to the Duncan test (α < 0.05).

Regarding plant height, environmental conditions in La Unión and Sabanagrande promoted greater growth compared with Sabanalarga. With few exceptions, growth in La Unión tended to surpass that in Sabanagrande (Figure 4a). Interestingly, the environmental effects on first branching height did not mirror those on plant height, particularly for SM-28 and SM-22 (Figure 4b). The highest production of planting material (number of stakes per plant) occurred in La Unión and Sabanagrande, corresponding to the Humid and Dry Caribbean subregions, respectively (Figure 4c). As with plant height, Sabanalarga exhibited the lowest number of stakes per plant.

In terms of yield, the analysis of variance indicated that location (L) had the strongest and most significant influence on commercial root weight per plant (Table 5). Although genotype (G), age at harvest (Age), and their interactions (L × G and L × Age × G) were also significant, their contributions to the total variance were considerably smaller than that of the environment.

For FRY, DMC, DRY, and ROT, location had a significant effect and accounted for a large proportion of the observed variation (Table 5). Genotype also contributed significantly to the variability in all response variables, as did age at harvest. The interactions L*G and L*Age were significant for all traits analyzed. In contrast, the Age*G interaction was not significant for ROT.

**Figure 5:**
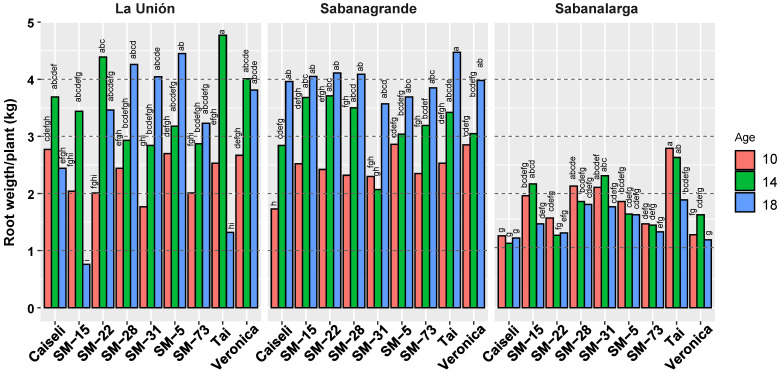
Response of weight of commercial roots (kg/plant) in nine genotypes evaluated under different environmental conditions (La Unión, Sabanagrande and Sabanalarga) and ages at harvest time (10, 14 and 18 months after planting). Means followed by a common letter (within each location) are not significantly different according to the Duncan test (α < 0.05).

The environmental conditions in La Unión and Sabanagrande (Humid Caribbean subregion) positively influenced root production per plant, resulting in higher yields. In contrast, conditions in Sabanalarga (Dry Caribbean subregion) were unfavorable, leading to the lowest root weight per plant (Figure 5).

Overall, there was a clear trend of increasing yield per plant across harvest ages (Figure 5). With one exception (SM-31), productivity in the Humid Caribbean subregion of Sabanagrande consistently increased with harvest age. In the second Humid Caribbean location (La Unión), yields increased from 10 to 14 MAP in all genotypes; however, continued increases through 18 MAP were observed only in SM-28, SM-31, SM-5, and SM-73. From 14 to 18 MAP, productivity declined slightly in Verónica, more noticeably in SM-22 and Caiseli, and sharply in SM-15 and TAI. In the Dry Caribbean subregion (Sabanalarga), productivity was substantially lower than in the other locations, with no clear response to harvest age (Figure 5).

**Figure 6:**
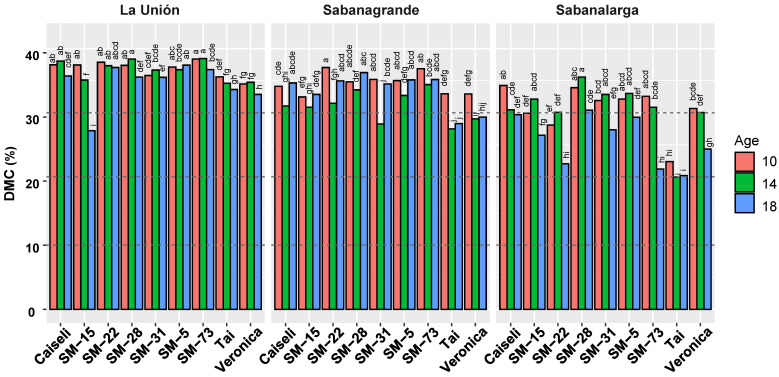
Response of dry matter content in roots from nine genotypes evaluated under different environmental conditions (La Unión, Sabanagrande and Sabanalarga) and ages at harvest time (10, 14 and 18 months after planting). Means followed by a common letter (within each location) are not significantly different according to the Duncan test (α < 0.05).

Figure 6 summarizes the DMC responses for each location separately, given the strong interactions among genotype, harvest age, and environment. TAI, the regional commercial reference for industrial processing due to its overall productivity, revealed its main limitation: a below-average DMC, which declined further at the extended harvest ages, especially in Sabanagrande and Sabanalarga. Caiseli, the reference variety for its consistently high DMC, confirmed this characteristic in the present study. However, its DMC decreased sharply at Sabanalarga when harvest was extended.

The experimental clone SM-28 showed not only competitive DMC levels compared with the reference variety Caiseli, but also remarkable stability (Figure 6). Differences in DMC between 10 and 14 MAP were not statistically significant. Moreover, DMC values at La Unión and Sabanalarga increased at 14 MAP, suggesting a rapid recovery following the onset of the rainy season. At 18 MAP, DMC varied across locations, reaching its highest level in Sabanagrande and its lowest—significantly so—in La Unión, with a similarly low value in Sabanalarga. Clone SM-31 also displayed high and stable DMC levels, except at 14 MAP in Sabanagrande and 18 MAP in Sabanalarga.

### 3.4. Variety Selection Among Six Experimental Genotypes Under Delayed Harvest

The relative performance of SM-28 (expressed as percentage increase or decrease) in FRY, DMC, and DRY compared with the three commercial controls is shown in Table 6. Although SM-28 did not surpass Tai in FRY, its 23% higher DMC resulted in an overall increase of more than 6% in DRY. Relative to Verónica, SM-28 showed increases in both FRY and DMC, leading to a 24% gain in DRY. Finally, compared with Caiseli, SM-28 demonstrated substantial improvements in FRY (27%) and DMC (5%), yielding an increase of more than 30% in DRY across the three harvest ages.

A selection index was developed using FRY with a weight of 10, DMC with a weight of 12, and PTS with a weight of –5 (Table 7). The analysis across environments (location × harvest age) showed that genotypes SM-28, SM-5, and SM-73 had significantly higher average performances (12.51, 4.38, and 7.05, respectively). Among these, only SM-28 demonstrated superiority in eight environmental conditions. The remaining experimental genotypes showed superiority in only one to four conditions, with negative average selection index values. The only commercial clone with a (marginally) positive average index was Caiseli.

## 4. Discussion

Extended harvests—up to 16 months after planting (MAP)—have been successfully implemented in southern Brazil [29,30]. Lengthening the growth cycle enables a second harvest season, and although this approach requires a few additional field operations, the gains are substantial, and fresh root yield (FRY) can double compared to standard harvest times. A critical prerequisite for this strategy, however, is that cultivars must be able to maintain or restore their DMC after the onset of rains (or rise in temperatures after winter in Southern Brazil), which stimulate foliar regrowth.

DMC is strongly influenced by environmental conditions. In this study, two cycles of selection were conducted in which genotypes were evaluated under delayed harvest conditions. As expected, a sharp decline in DMC was observed when plants were harvested after the onset of the first rains following the dry season. However, this reduction was not uniform across genotypes. In contrast to commercial check varieties, genotypes such as SM-5, SM-31, SM-28, SM-73, SM-22, and SM-15 showed a rapid recovery of DMC, demonstrating clear adaptation to delayed harvests at ≥15 MAP. These results suggest that DMC recovery after delayed harvest is at least partially under genetic control and can therefore be enhanced through breeding [20,31]. OMICs approaches could further contribute to understanding the genetic and physiological pathways underlying DMC stability and facilitate the implementation of genomic selection strategies [32,33].

A selection index incorporating FRY, PTS, and DMC identified six genotypes (SM-5, SM-31, SM-28, SM-73, SM-22, and SM-15) to be evaluated in the second phase. These genotypes consistently showed excellent FRY, PTS, and exhibited both high and good DMC recovery under extended harvest conditions. Consistent with previous studies, the selection index is a useful methodology, as combined analyses across environments show that genotypes with positive selection index values consistently exhibit good agronomic performance in most environments [14,34].

The most cultivated variety in the Caribbean Region of Colombia is Tai (or Rayong 60 in Thailand). This clone showed good FRY and excellent PTS, but its DMC was generally low and failed to recover under extended harvest conditions as reported elsewhere [7,8]. Reflecting the commonly observed negative relationship between DMC and FRY [13], clone SM-42 achieved the highest average DMC (35.25%) but produced a relatively low FRY (14.82 t/ha). DMC and FRY are strongly influenced by genetic factors, with non-additive genetic effects playing a particularly important role in FRY [35].

The multivariate analysis revealed that plant height, height of the first branch, and the number of branching events, key components of the PTS, are closely linked and contribute substantially to the variability captured by the first principal component. Because lower PTS values indicate better plant architecture, the direction of these correlations is consistent with expectations. The height of the first branch and the number of branching events were negatively correlated. As the first branching occurs higher on the plant, the total number of branches tends to decrease, resulting in a more agronomically desirable architecture [9]. An erect plant architecture facilitates cultural practices and their mechanization and extends the viability of stored stems. Plant architecture is strongly influenced by genetic factors, and taller plants do not necessarily exhibit a higher first branching height [12,15,36,37].

The second phase of selection in the agronomic evaluation of the six experimental genotypes selected, showed that plant height, height of first branching, and stakes per plant were primarily influenced by location and genotype, with age at harvest contributing to a lesser extent. During the extended harvest, plants continued to grow, reaching their maximum plant height and first branching height at 18 MAP. By this time, however, the quality of planting material had declined, which explains why a greater number of stakes per plant was recorded at 10 MAP. Regarding plant height, environmental conditions in La Unión and Sabanagrande promoted greater growth compared with Sabanalarga. These results are consistent with previous studies that showed the environmental conditions impact on plant architecture [14].

In cassava, traits such as FRY, DMC, and plant architecture are strongly affected by the environment and by genotype-by-environment interactions [38,39]. In terms of yield, the analysis of variance indicated that location had the strongest and most significant influence on commercial root weight per plant, FRY, DMC, DRY, and ROT. Although genotype, age at harvest, and their interactions were also significant, their contributions to the total variance were considerably smaller than that of the environment. There was a clear trend of increasing yield per plant across harvest ages, however it was environment-dependent. The magnitude of this influence varies considerably among genotypes [30,40].

Cassava breeding has made substantial contributions through the release of improved varieties since the 1990s, achieving higher fresh root yield (FRY) and increased dry matter content (DMC) [8,16,17]. Although identifying genotypes with high DMC or high FRY is relatively straightforward, finding those that excel in both traits simultaneously remains a major challenge [19,35]. SM-28 combines competitive FRY—comparable to commercial checks—with superior and remarkably stable DMC. The identification of SM-28 represents a significant contribution to Colombia’s industrial cassava value chain and provides encouraging evidence that DMC stability is genetically controlled and can be enhanced through breeding.

The relative performance of SM-28 (expressed as percentage increase or decrease) in FRY, DMC, and DRY compared with the three commercial controls shows that, although SM-28 did not surpass Tai in FRY, its 23% higher DMC resulted in an overall increase of more than 6% in DRY (Table 6). DRY wise, SM-28 also showed higher productivity compared with Caiseli and Veronica checks. In the present work, the inclusion of harvest age added further complexity to the analysis and comparison of genotypes due to the strong interactions associated with this factor.

Finally, results from this study indicate that the operational period of processing industries can be extended through the use of improved cassava varieties. In Brazil, it was shown that FRY can increase by 29% with extended growing cycles, although DMC decreases by 6.28%. Despite this reduction, overall productivity—and thus farmer profitability—can still increase, while the processing industry benefits from an expanded operational capacity [29].

### Future Prospect

Future efforts should focus on improving experimental designs to reduce error and enhance precision in the phenotyping process. The identification of genotypes that clearly contrast in their capacity to recover DMC reported in the present study could facilitate the development of molecular markers and improve our understanding of the genetic and physiological factors influencing this trait. The genotype SM 2828-28 identified during these evaluations could be the first variety registered as improved variety suitable for extended harvest for Caribbean region in Colombia. Finally, this study also revealed the logistical complexities inherent in research involving extended harvest ages. Strong interactions among the different sources of variation complicated the statistical analysis and obscured genetic differences. These challenges should be carefully considered in future research on this topic.

## 5. Conclusions

These results contribute to efforts to extend the harvest period and mitigate production seasonality in Colombia. In most current commercial cassava varieties, dry matter content (DMC) declines under extended harvest after the onset of the rainy season and does not subsequently recover. In contrast, our results identified an improved cassava variety (SM 2828-28) combining high fresh root yield (FRY) and, most importantly, consistently stable DMC over time. This stability in DMC would provide producers with greater confidence in securing favorable prices for their roots and enable processors to achieve higher production efficiency [9].

This study underscores the importance of DMC stability as a critical trait for addressing the challenges posed in cassava by climate change. Moreover, the evidence indicates that DMC stability is under genetic control, suggesting that it can be effectively enhanced through targeted breeding efforts.

## Figures and Tables

**Table 1:** Evaluated genotypes and their pedigree.

N°	Genotype	ID	Progenitor(s)
1	GM214-62	GM-62	CM 523-7(COL 655A × COL 1515) × SM 1411-5(GG 1141-1 × ?)
2	GM579-13	GM-13	SM 1789-20(COL 1505 × ?) × CM 8027-3(CM 3555-6 × MAL 2)
3	SM2629-36	SM-36	SM 1422-4 (CM 3306-4 × ?) × ?
**4**	**SM2828-28**	**SM-28**	**CM 7389-9 (MCOL 2253 × CM 2177-2)**
**5**	**SM2834-31**	**SM-31**	**SM 1411-5 (GG 1141-1 × ?)**
**6**	**SM3110-15**	**SM-15**	**SM 1669-7 (SG 731-4 × ?)**
**7**	**SM3134-5**	**SM-5**	**SM 1438-2 (MTAI 8 × ?)**
**8**	**SM3134-73**	**SM-73**	
9	SM3135-14	SM-14	SM 2545-20 (SM 737-38 × ?) × ?
10	SM3135-30	SM-30	
11	SM3137-40	SM-40	SM 2546-24 (SM 890-9 × ?) × ?
**12**	**SM3139-22**	**SM-22**	**SM 2618-16 (CM 7389-9 × ?)**
13	SM3140-2	SM-2	SM 2619-1 (CM 7395-5 × ?) × ?
14	SM3144-42	SM-42	SM 2619-6 (CM 7395-5 × ?) × ?
15	SM3150-17	SM-17	CM 6754-8 (COL 2215 × CM 507-37) × ?
16	CORPOICA Costeña	Costeña	MMEX 11 × MCOL 65
17	CORPOICA Tai	TAI	MCOL 1684 × Rayong 1
18	CORPOICA Veronica	Verónica	MCOL 2207 × SM 301-3
19	CORPOICA Caiseli	Caiseli	SM 1278-2 (MMEX 11 × MCOL 65) × ?)

The experimental clones originated from the cassava improvement program at the International Center for Tropical Agriculture (CIAT) and were later transferred to AGROSAVIA for final selection and eventual release. After the initial evaluation, six experimental genotypes—SM2828-28, SM2834-31, SM3110-15, SM3134-5, SM3134-73, and SM3139-22 (highlighted in bold in Table 1)—were selected for further multi-site testing under Caribbean conditions.

**Table 2:** Agroecological zones used in the agronomic evaluations in this study (2017–2019).

Subregion	Department	Municipio	Harvest Time (MAP)
UYT			
Sub-Humid	Cordoba	Cienaga de Oro	12
Sub-Humid	Cordoba	Cerete	5-15
Dry	Sucre	Corozal	8-10-14-16-17-18
Sub-Humid	Sucre	La Union	13-15-16-17
Dry	Sucre	San Antonio de Palmito	10-15-16
Dry	Sucre	Bremen	12-15-18
Dry	Atlántico	Santo Tomas	8-9-10-11-12-14-15-16-17-18
AET			
Sub-Humid	Atlantico	Sabanagrande	10-14-18
Dry	Atlantico	Sabanalarga	10-14-18
Sub-Humid	Sucre	La Unión	10-14-18

**Table 3:** Summary of the evaluation across 39 trials of 15 experimental genotypes and 5 commercial checks under delayed harvests.

Clon	N	FRY	DRY	DMC	Root Rotting
		(t/ha)		(%)	(1–5 Scale)
SM-5	169	27.06 a	9.60 a	35.08 ab	0.61 g
SM-31	177	24.731 bc	8.59 bc	34.39 cd	0.73 fg
SM-28	170	23.51 c	8.12 ced	34.36 de	0.65 fg
SM-73	172	20.89 de	7.35 gh	34.77 bc	0.65 fg
SM-22	167	21.18 de	7.43 fgh	34.60 cd	0.75 efg
SM-15	176	24.41 bc	8.16 cd	33.22 g	0.99 edf
SM-36	167	27.26 ª	8.79 b	32.27 i	1.10 ed
SM-17	171	23.72 bc	7.96 edf	33.26 g	0.91 efg
GM-13	168	25.17 b	8.25 bcd	32.54 hi	0.79 efg
GM-62	175	24.32 bc	7.93 edf	32.69 h	0.88 efg
SM-40	162	20.84 de	7.20 gh	34.29 de	1.29 cd
SM-14	176	18.96 gf	6.44 j	33.47 g	0.56 g
SM-2	166	21.88 d	7.59 efg	33.89 f	1.98 b
SM-30	174	17.51 g	6.14 j	34.32 de	1.32 cd
SM-42	163	14.82 h	5.30 k	35.25 a	2.50 a
Verónica	121	19.94 ef	6.32 j	31.06 k	0.60 g
Ginés	116	24.19 bc	6.98 hi	29.59 L	0.88 efg
Caiseli	95	17.80 g	6.17 j	34.07 ef	2.64 a
Costeña	158	20.38 de	6.57 ij	31.71 j	1.64 bc
Tai	156	24.40 bc	7.28 gh	29.72 L	1.89 b

N: data number, FRY: Fresh Root Yield, DRY: Dry Root Yield, DMC: Dry Matter Content.

**Table 4:** Analysis of variance of plant architecture and growth in AET of nine cassava genotypes evaluated in three locations and harvested 10, 14 and 18 months after planting.

Source of Variation	df	Plant Height	Height 1st Branching	Stakes/Plant
		(cm)	(cm)	(Number)
Location (L)	2	223037.6 ***	4422.0 *	328.8 ***
AGE (A)	2	48623.5 ***	11513.6 ***	7.1 *
Genotype (G)	8	12313.4 ***	54633.9 ***	9.6 ***
L × A	4	4248.8 ***	1418.3	60.9 ***
L × G	16	3202.2 ***	13521.4 ***	9.6 ***
A × G	16	1110.1 **	2172.0 *	2.2
L × A × G	32	517.5	557.1	1.4
Residuals	162	508.6	927.8	1.5

*, **, and *** show significant differences at *p* < 0.01, 0.001, and 0.0001, respectively. Df: degree of freedom. The table presents mean square values.

**Table 5:** Mean square values from the analysis of variance for weight of commercial roots per plant (WCR/P), fresh root yield (FRY), dry matter content (DMC), dry root yield (DRY) and rotten roots (ROT) in agronomic tests of nine cassava genotypes (G) evaluated in three locations (L) and harvested 10, 14 and 18 months after planting (Age).

Source of Variation	df	WCR/P	FRY	DMC	DRY	ROT
Location (L)	2	52.5 ***	6638.9 ***	991.3 ***	1284.2 ***	1459.5 ***
Age	2	11.5 ***	1404.5 ***	133.6 ***	179.1 ***	362.4 ***
Genotype (G)	8	1.1 *	328.6 ***	112.4 ***	28.2 **	678.6 ***
L × Age	4	7.9 ***	854.1 ***	92.4 ***	127.6 ***	195.3 ***
L × G	16	1.3 ***	204.4 ***	31.1 ***	27.2 ***	331.3 ***
Age × G	16	1.1 ***	106.6 *	7.1 ***	12.0 *	40.2
L × Age × G	32	0.9 ***	102.1 *	7.1 ***	13.5 *	41.8 *
Residual	162	0.4	51.7	1.8	6.9	24.2

*, **, and *** show significant differences at *p* < 0.01, 0.001, and 0.0001, respectively. Values show mean squares data for each variable. Df: degree of freedom, WCR/P: weight of commercial roots per plant, FRY: Fresh Root Yield, DMC: Dry Matter Content, DRY: Dry Root Yield, ROT: Root rot.

**Table 6:** Percentage of increase or reduction in fresh root yield, dry matter content and dry root yield between the genotype SM2828-28 and the three commercial controls.

Commercial Check	Fresh Root Yield (%)	Dry Matter Content (%)	Dry Root Yield (%)
Corpoica Tai	−11.96	23.12	6.27
Corpoica Verónica	10.79	13.42	23.58
Corpoica Caiseli	26.94	5.45	30.2

**Table 7:** Selection index obtained by integrating fresh root yield, dry matter content, and plant type for different environments (seasons × location). Green indicates genotypes with a selection index above average (superior performance), gray shows values slightly above or below average, and red shows strong negative values.

Environment	SM-28	SM-5	SM-73	SM-15	SM-31	SM-22	TAI	Veronica	Caiseli
Sabanagrande-10	1.67	−7.23	16.58	−8.95	−7.78	20.48	1.70	0.18	−16.64
Sabanagrande-14	20.68	−6.43	19.93	5.09	−18.83	−7.80	4.19	−12.20	−4.63
Sabanagrande-18	17.83	−2.86	10.38	4.00	−9.17	0.13	1.33	−23.06	1.41
Sabanalarga-10	13.09	9.06	2.69	0.98	3.54	−13.31	−11.10	−7.10	2.15
Sabanalarga-14	19.31	7.46	−0.59	11.30	3.68	−7.54	−9.60	−1.54	−22.48
Sabanalarga-18	18.55	19.71	−15.41	−0.05	10.00	−18.39	−12.27	−5.82	3.68
La Unión-10	6.14	6.69	3.49	−13.80	−22.00	−0.44	3.78	−4.42	20.55
La Unión-14	5.00	−1.03	9.25	−19.95	−12.66	0.36	5.72	0.55	12.74
La Unión-18	10.31	14.02	17.10	−44.18	3.51	0.10	−15.25	0.17	9.13
Average	12.51	4.38	7.05	−7.28	−5.52	−2.93	−3.50	−5.92	0.66
Sup. performance	8	5	7	3	4	1	3	0	5

## Data Availability

All the relevant data are available in the manuscript itself. Additional data can be provided on request.
